# Molecular genotyping and subgenotyping of duck circovirus at duck farms in Thailand

**DOI:** 10.14202/vetworld.2024.1990-1999

**Published:** 2024-09-08

**Authors:** Sittinee Kulprasertsri, Thaweesak Songserm, Sakuna Phatthanakunanan, Pattrawut Saengnual, Nuananong Sinwat, Raktiphorn Khamtae, Preeda Lertwatcharasarakul

**Affiliations:** 1 Department of Farm Resources and Production Medicine, Faculty of Veterinary Medicine, Kasetsart University, Kamphaeng Saen, Nakhon Pathom, Thailand; 2 Department of Pathology, Faculty of Veterinary Medicine, Kasetsart University, Kamphaeng Saen, Nakhon Pathom, Thailand; 3 Kamphaeng Saen Veterinary Diagnostic Center, Faculty of Veterinary Medicine, Kasetsart University, Kamphaeng Saen, Nakhon Pathom, Thailand

**Keywords:** duck, duck circovirus, genetic characterization, immunosuppression, phylogenetic tree

## Abstract

**Background and Aim:**

Ducks worldwide are infected with duck circovirus (DuCV), which causes feather abnormality, emaciation, and poor growth performance. DuCV is similar to other circoviruses that induce immunosuppression due to the occurrence of the bursae of Fabricius (BF) and spleen atrophies. In Thailand, retarded ducks with feather losses were submitted for disease investigation. The ducks presented low body weight gain, had small BF and spleens, and were consistent with duck-infected DuCV. Our study investigated the possibility of DuCV infection in duck flocks in Thailand. We also analyzed the genetic characteristics of the virus.

**Materials and Methods:**

BF and spleen samples were collected from affected meat and layer ducks from six farms thought to have been infected with DuCV. These tissues were then subjected to histopathological examination and molecular identification using conventional polymerase chain reaction and nucleotide sequencing. To identify DuCV, phylogenetic trees were generated using MEGA version X software. Samples of tissues or swabs were collected to determine whether coinfections with bacteria and viruses existed.

**Results:**

Phylogenetic analysis using the entire genome (1995–1996 bp) and *cap* gene (762 bp) revealed that the DuCV isolates circulating in Thailand belonged to DuCV genotype I, which was further subdivided into two sub-genotypes: sub-genotype I b and an unclassified sub-genotype based on reference sub-genotypes. Thai isolates have variations in 10 amino acid residues in the capsid protein. Ducks infected with Thai DuCV were also coinfected with *Riemerella anatipestifer*, *Escherichia coli*, *Pasteurella multocida*, duck viral enteritis, and duck Tembusu virus, which is consistent with previous DuCV infection studies.

**Conclusion:**

Six DuCVs from ducks who were previously found to have feather loss, were underweight, had growth retardation, and had poor body condition were identified in this study as belonging to genotype I and constituting at least two sub-genotypes. Due to the immunosuppressive effects of DuCV, coinfection of bacterial and viral pathogens was typically observed in Thai DuCV-infected ducks.

## Introduction

Duck circovirus (DuCV) causes feathering disorders, delayed growth, and decreased body weight [1, 2]. An experimental study reported that DuCV-infected ducks exhibit smaller immune organ size than non-infected ducks [3]. Histopathological examination of the bursae of Fabricius (BF) revealed lymphoid depletion, necrosis, and histiocytosis [1, 2]. Hong *et al*. [1] reported that DuCV induces immunosuppression in response to severe immune organ damage. Therefore, DuCV-infected ducks can be easily susceptible to secondary infections such as *Riemerella anatipestifer* (RA), avian pathogenic *Escherichia coli* (APEC)*, Pasteurella multocida*, *Salmonella* Enteritidis, and duck hepatitis virus 1 (DHV-1), resulting in increased morbidity and mortality rates with the threat of severe economic losses [3, 4]. DuCV, first detected in Germany from Mulard ducks in 2003, belongs to the genus *Circovirus*, a subfamily of the *Circoviridae* family [5]. The structure of DuCV is similar to that of other circoviruses under an electron microscope [6]. The virion is small, non-enveloped, icosahedral, 15–16 nm in diameter [7]. The genome of DuCV is a single-stranded circular DNA of approximately 1.99 kb that contains three major open reading frames (ORFs), including ORF-1, ORF-2, and ORF-3 [5, 8, 9]. ORF-1 encodes the replication protein (Rep). ORF-2 encodes the major immunogenic capsid protein (Cap) [5]. ORF-3 is located on the complementary strand of ORF-1 and encodes the ORF-3 protein, which is a novel protein with an apoptosis mechanism involved in DuCV pathogenesis [8, 9].

DuCV was previously characterized into two main genotypes (DuCV-1 and DuCV-2) based on the phylogenetic tree of the complete genome and *cap* gene [10, 11]. DuCV-1 is widely distributed in many countries, including Germany, Hungary, the USA, Poland, China, South Korea, and Vietnam, whereas DuCV-2 is found in Taiwan, China, and Vietnam [1, 12–14]. DuCV-3, with a genome size of 1755 nt, was defined based on the nucleotide and amino acid sequences of the virus isolated from layer ducks with a severe decrease in egg production in China [15]. Recently, DuCV has had the highest infection rate among Chinese breeder flocks, resulting in viral persistence in these flocks [16–18]. Li *et al*. [19] reported coinfection of DuCV-1 and DuCV-2 in 1-day-old ducklings, dead embryos, and unfertilized eggs, indicating vertical transmission of DuCV. Based on epidemiological studies, DuCV infections were highly prevalent in China (33.3%) [20], South Korea (21.8%) [20], Vietnam (43.08%) [20], Hungary (84%) [21], Taiwan (38.2%) [22], and Germany (46%) [22, 23]. There is evidence that the virus is widespread in the duck industry worldwide [4, 24].

Previous studies have indicated that individual circovirus infection induces subclinical poor animal growth performance [3, 4]. The subclinical infection of DuCV is challenging to diagnose, followed by latent infection, which potentially poses long-term economic losses for the commercial duck industry. Hence, circovirus-induced immunosuppression in ducks is an essential global concern [3]. In South-east Asia, DuCV was first isolated from weak and deficient ducks in Vietnam. Vietnamese DuCV isolates are classified into two genotypes and several sub-genotypes [20]. In addition, Thailand is the tenth most duck-producing country in the world (http://www.trademap.org/). At present, low-weight retarded ducks are present in many duck farms in the central and eastern regions of Thailand. The clinical symptoms of the affected ducks were similar to those of previous DuCV outbreaks.

Hence, the objective of this study was to investigate DuCV infection in duck flocks in Thailand and to study the genetic characteristics of DuCV.

## Materials and Methods

### Ethical approval

Kasetsart University Institutional Animal Care and Use, Thailand (ACKU65-VET-076, September 20, 2022), approved all procedures involving animal care and use for scientific research.

### Study period and location

The study was conducted from October 2022 to October 2023 at Kamphaeng Saen Veterinary Diagnostic Center, Faculty of Veterinary Medicine, Kasetsart University, Kamphaeng Saen Campus.

### Sample collection

Six duck farms with moderate-to-high culling rates submitted sick ducks for necropsy at the Kamphaeng Saen Veterinary Diagnostic Center. Affected ducks, including meat and layer ducks, were raised in closed farming or free-grazing systems. The age of the ducks ranged from 5 to 27 weeks and originated from duck farms located in the Nakhon Pathom (NK), Chachoengsao (CCS), Ratchaburi (RB), and Suphan Buri (SP) provinces of Thailand. For molecular DuCV detection, spleen and BF tissues were collected from 96 ducks from six farms. For coinfection investigation, additional organs associated with possible bacterial or viral infections were gathered.

### Histopathological preparation

The tissue samples were fixed in 10% neutral buffered formalin solution, dehydrated, embedded in paraffin blocks, sectioned to 5 μm thickness, and stained with hematoxylin and eosin (H&E) using a standard protocol.

### Bacterial culture and identification

To test for bacterial coinfection, sinus swabs or tissues associated with fibrinous inflammation were collected for bacterial culture and identification. The collected samples were inoculated on MacConkey agar and blood agar (trypticase soy agar mixed with 10% sheep blood) and then incubated for 24–48 h under appropriate conditions depending on the presumptive bacteria. Bacterial colonies were tentatively confirmed by Gram-staining and biochemical tests.

### DNA extraction and purification

The same flock’s tissue was pooled, and DNA and RNA were extracted using the IndiMag Pathogen (Indical BioScience, Germany) extraction kit according to manufacturing recommendations. Spleen and BF DNA samples were collected using the polymerase chain reaction (PCR) to determine the presence of DuCV. A PCR was performed using intestine DNA samples to detect duck viral enteritis virus (DVEV) coinfection. Finally, brain and spinal cord RNA samples were analyzed by reverse transcription (RT)-PCR to identify duck tembusu virus (DTMUV).

### PCR

The whole genome of DuCV was assembled from overlapping amplicons and subjected to nucleotide sequence analysis by PCR using two primer pairs [25]. A DreamTaq Green PCR Master Mix (ThermoFisher Scientific, Lithuania) was used to amplify the target viral gene. 20 μL reaction contained 10 μL of 2× DreamTaq Green PCR Master Mix, 0.2 μL of each primer (final concentration 10 μM), 7.6 μL nuclease-free water, and 2 μL of extracted total DNA. The PCR conditions consisted of pre-denaturation at 95°C for 3 min, 40 cycles of denaturation at 95°C for 30 s, primer annealing at 55°C for 30 s, and extension at 72°C for 1 min, and final extension at 72°C for 5 min. The amplified DNA target was analyzed by 1.5% agarose gel electrophoresis to confirm the length of the amplified fragments. Each amplicon was purified using FavorPrep GEL/PCR Purification (Favorgen, Taiwan), and nucleotide sequences were analyzed by NGS using the MiSeq Illumina sequencing platform at BTSeq^ΤΜ^ Contiguous Sequencing Service (Celemics, Seoul, South Korea). For DTMUV and DVEV detection, RT-PCR and PCR protocols were set up as previously described by Su *et al*. [26] and Hansen *et al*. [27].

### Nucleotide sequencing and phylogenetic analysis

The nucleotide sequences were identified as organisms using the nucleotide Blast program (https://blast.ncbi.nlm.nih.gov/Blast.cgi). Multiple nucleotide alignments among the nucleotide sequences of our isolates and other isolates obtained from GenBank database (https://www.ncbi.nlm.nih.gov/nucleotide) were performed using ClustalW program in MEGA version X software [28]. Genetic distances were calculated using the Kimura 2-parameter method. The neighbor-joining method generated a phylogenetic tree with 1000 bootstrap replicates [28]. The general information and accession numbers of all Thai DuCV isolates (accession number OQ744001-OQ744006) and reference isolates obtained from GenBank are presented in Table-1.

**Table-1:** Information about Thai DuCV isolates, reference genotypes, and sub-genotypes from GenBank database.

Host	Collection Year	Isolates	Country	Sub-genotype	Accession number
Cherry Valley Duck	2022	DuCV_NK_KU001-22	Thailand	This study	OQ744001
Layer Duck	2022	DuCV_NK_KU003_22	Thailand	This study	OQ744002
Layer Duck	2022	DuCV_NK_KU005_22	Thailand	This study	OQ744003
Cherry Valley Duck	2022	DuCV_CCS_KU002_22	Thailand	This study	OQ744004
Layer Duck	2022	DuCV_SP_KU004_22	Thailand	This study	OQ744005
Layer Duck	2022	DuCV_RB_KU006_22	Thailand	This study	OQ744006
Duck	2019	HN07	China	Ia	MN928801
Cherry Valley Duck	2017	YF180401	China	Ia	MN068357
Duck	2021	Vietnam/VNUA-HN47/2021	Viet Nam	Ia	OM176553
Duck	2021	Vietnam/VNUA-TB61/2021	Viet Nam	Ia	OM176554
Cherry Valley ducks	2021	DuCV QD	China	Ia	MZ425925
Duck	2021	Vietnam/VNUA-HY40/2021	Viet Nam	Ib	OM176552
Duck	2011	D11-JW-022	South Korea	Ib	KC851810
Duck	2011	D11-JW-024	South Korea	Ib	KC851811
Duck	2011	D12-KD-019	South Korea	Ib	KC851819
ND	2012	NN12/2012	China	Ic	KC460531
Duck	2021	Vietnam/VNUA-TN85/2021	Viet Nam	Ic	OM176555
Anas platyrhynchos	2014	VC4	Brazil	Ic	MT318126
Pekin duck	ND	33753-52	USA	Ic	NC007220
Cherry Valley Pekin ducks	2015	SDFC12	China	Ic	KY328304
Cherry Valley ducks	2016	JSPX03E	China	Ic	MF627688
Muscovy duck	2009	LZ/11/09	China	Id	HQ180265
Mulard duck	ND	DuCV	Germany	Id	AY228555
Cherry Valley Duck	2008	WF0801	China	Id	GU131340
Muscovy duck	ND	MH25	China	Id	EF451157
M18 Mule duck	ND	MH02/07	China	IIa	EU499309
Muscovy duck	ND	MH11	China	IIa	EU344805
Muscovy duck	ND	HZ09	China	IIa	EU344802
Muscovy duck	ND	TC1/2002	Taiwan, China	IIb	AY394721
Muscovy duck	ND	TC2/2002	Taiwan, China	IIb	DQ166836
Muscovy duck	ND	TC3/2002	Taiwan, China	IIb	DQ166837
Muscovy duck	ND	TC4/2002	Taiwan, China	IIb	DQ166838
Muscovy duck	2012	CP12021	Taiwan, China	IIb	KP229377
Duck	2021	Vietnam/VNUA-BG135/2021	Viet Nam	IIb	OM176557
Muscovy duck	2008	WS-GD01	China	IIc	FJ554673
Muscovy duck	2006	FJ0601	China	IIc	EF370476
Duck	2007	LY0701	China	IIc	EU022374
Cherry Valley Duck	2008	WF0804	China	IIc	GU131343
Duck	2021	Vietnam/VNUA-HD89/2021	Viet Nam	IIc	OM176556
Cherry Valley Duck	2018	YN180506	China	IIc	MK814585
Muscovy duck	2009	FJFQ315	China	IIc	GQ423744
Duck	2022	HNU-HYH-2022	China	III	OP432310

ND=No data, DuCV=Duck circovirus

## Results

### Clinical observations of ducks infected with DuCV and other spp.

**Figure-1:**
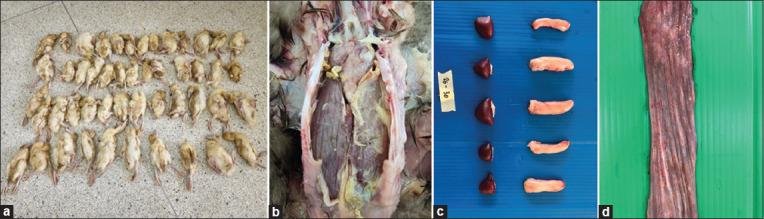
Clinical findings of ducks with various duck diseases and DuCV infections in Thailand. (a) Meat ducks infected with DuCV presented with non-uniformity, stunted growth, and feathering disorders. (b) Ducks coinfected with DuCV and *Riemerella anatipestifer* frequently presented with fibrinopurulent polyserositis. (c) Various sizes of bursae of Fabricius and spleen collected from meat ducks coinfected with DuCV and RA. (d) Layer ducks infected with DuCV showed hemorrhagic and multifocal necrosis of the esophageal mucosa, which was related to duck viral enteritis virus coinfection. DuCV=Duck circovirus.

All duck farms (6/6) typically presented with feather losses, lower-than-average weight, growth retardation, and poor body condition, consistent with the clinical signs of DuCV infection in previous studies (Figure-1a). In this study, almost all cases (4/6) of DuCV infection presented with fibrinopurulent polyserositis suspected to be associated with RA coinfection (Figure-1b). In this study, it was noticed that there was a significant variation in the sizes of the spleen and BF at all farms (6/6), as shown in Figure-1c. In a duck flock located in the SP province, a combination of DuCV, DTMUV, and RA infection was observed. The affected ducks exhibited severe neurological signs and fibrinous polyserositis. One-layer flock with a history of high mortality presented with generalized whitish-to-creamy nodules in the body cavity and hemorrhagic necrosis with a diphtheritic membrane in the gastrointestinal tract, which were referred to as fungal and DVEV infections, respectively (Figure-1d). The causative agents in this case were identified based on the presence of fungal hyphae and granulomatous inflammation in H&E slides (1/6) and a positive PCR test for DVEV infection (1/6). Layer ducks from one farm in the NK province presented with unilateral sinusitis and mucopurulent discharge, indicating bacterial infection associated with pasteurellosis, as confirmed by bacteriological analysis (1/6).

### Histopathological findings

Histopathological findings of immune organs obtained from all farms showed massive destruction of lymphoid cells in both the spleen and BF. Focal necrosis of lymphocytes and infiltration of mononuclear cells were observed in BF samples collected from DuCV-infected ducks.

### Genetic analysis

Positive samples were collected from six farms in four Thai provinces. Three samples were collected from three farms in NK, and the remaining samples were collected from farms in RB, CCS, and SP. All nucleotide sequences of the samples were confirmed as DuCV using the nucleotide blast program. Three of the six isolates (DuCV CCS KU002-22, DuCV_SP_KU004_22, and DuCV_RB_KU006_22) had complete genomes measuring 1995 bp, whereas the remaining isolates had genomes measuring 1996 bp (DuCV NK KU001-22, DuCV NK KU003-22, and, DuCV NK KU005-22). The non-coding DNA region appeared to have a single position difference. Nucleotide sequence analysis showed that the Thai DuCV isolates had typical circovirus characteristics, including three significant ORFs: ORFV1 (Rep protein), ORFC1 (Cap protein), and ORFC2 (apoptosis-related protein). We also found a noncoding region, a stem ring structure.

**Figure-2:**
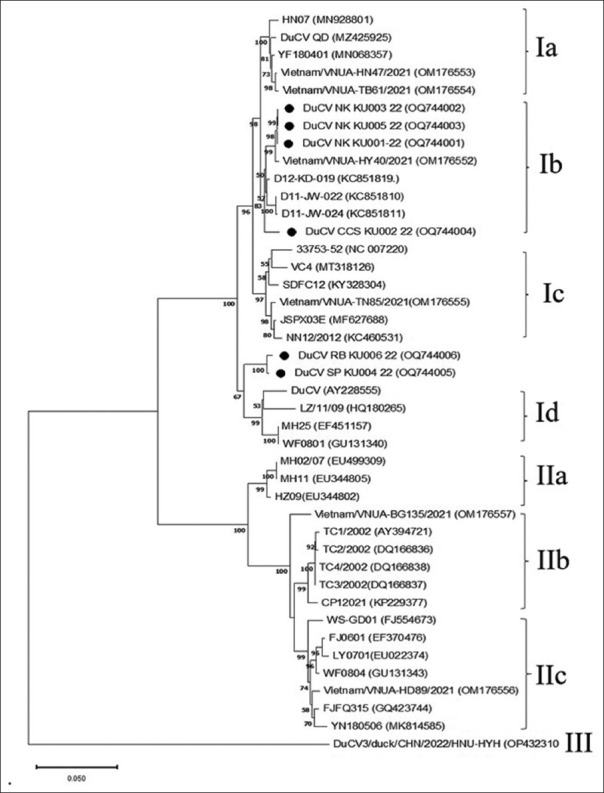
Phylogenetic tree based on complete genome sequences of Thai DuCV isolates and reference sequences from GenBank using neighbor-joining. Percentage of replicate trees in which the associated taxa were clustered together in the bootstrap test (1000 replicates) are shown above the branches. The tree is drawn to scale, with branch lengths in the same units as the evolutionary distances used to infer the phylogenetic tree. DuCV=Duck circovirus.

The complete genomes of our isolates and those obtained from GenBank were analyzed using multiple alignments and generated a phylogenetic tree (Figure-2). Our isolates were classified as genotype I (94.72%–99.53% similarity within reference genotype I isolates retrieved from the GenBank database) based on genetic distance and phylogenetic pattern; nevertheless, their isolates were further classified into two groups. The first group contained four Thai isolates (DuCV NK KU001-22, DuCV NK KU003-22, DuCV NK KU005-22, and DuCV CCS KU002-22), which had 98.52%–100% similarity within the group and were classified into sub-genotype Ib with 98.22%–99.53% similarity of reference sub-genotype Ib isolates. The second group consisted of two Thai isolates (DuCV RB KU006-22 and DuCV SP KU004-22), with 99.59% similarity. However, these isolates could not be classified into a reference sub-genotype because their similarity percentages were almost similar to Ia-Id reference sub-genotypes, ranging from 95.17% to 97.02% (Table-2).

**Table-2:** Percent similarity of our isolates within and between sub-genotypes based on complete genome and cap gene. DuCV NK KU001-22, DuCV NK KU003-22, and DuCV CCS KU002-22 isolates are included in group 1, and DuCV RB KU006-22 and DuCV SP KU004-22 isolates are included in group 2.

Group	Sequence location	Percent similarity				

		Within group	Between sub-genotypes			

			Ia	Ib	Ic	Id
Group 1	Complete genome (1996bp)	98.52–100	97-81–98.35	98.22–99.53	96.35–97.75	94.72–96.22
	*Cap* gene (762bp)	97.36–100	95.46–97.23	97.36–99.87	93.64–95.32	90.29–92.80
Group 2	Complete genome (1995bp)	99.59	96.16–96.83	96.16–97.02	95.17–96.47	95.23–96.59
	*Cap* gene (762bp)	99.09	90.01–91.91	90.43–92.20	88.81–91.04	92.50–94.06

DuCV=Duck circovirus

**Figure-3:**
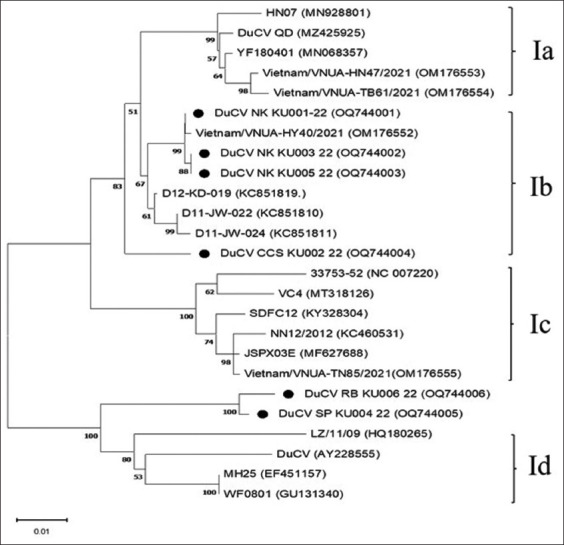
Phylogenetic tree based on complete *cap* gene sequences of Thai isolates and reference sequences available in GenBank using neighbor-joining. Percentage of replicate trees in which the associated taxa were clustered together in the bootstrap test (1000 replicates) are shown above the branches. The tree is drawn to scale, with branch lengths in the same units as the evolutionary distances used to infer the phylogenetic tree.

In all isolates, the *cap* gene was 762 bp long. In addition, our isolates were divided into two groups according to genetic distance and phylogenetic pattern, which resembled previously used complete genomes (Figure-3). Within group I, four samples exhibited 97.36%–100% similarity, with sub-genotype Ib exhibiting 97.36%–99.87% similarity. Due to the similarity percentage of the samples in group 2 being near the reference Ia-Id sub-genotypes, which were 90.01–91.91, 90.43–92.20, 88.81–91.04, and 92.50–94.06, respectively, subgenotype determination was not possible (Table-2).

**Figure-4:**
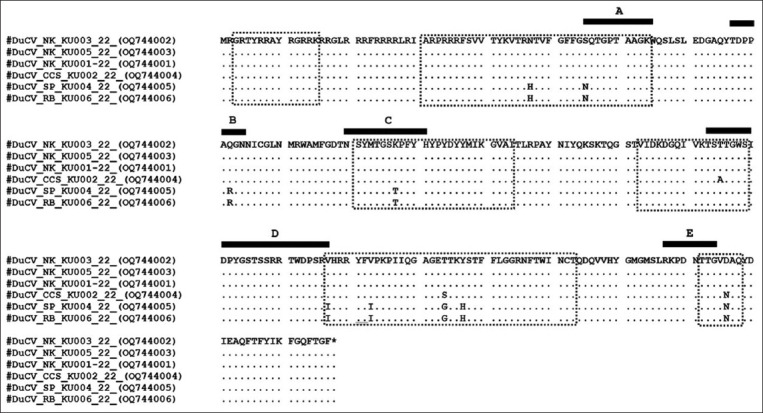
Amino acid alignments of the complete-coding Cap protein of six DuCVs isolated in Thailand. Black dots represent amino acids identical to those of DuCV_NK_KU003_22. Six major variable regions are indicated by rectangular dotted lines. Black lines represent A-E epitope regions.

Based on the 257 amino acid residues in Cap, genotype I contained 21 different amino acid residues, and 18 and 11 amino acid residues were in major variable and epitope regions, respectively (Table-3). All isolates from Thailand have alterations in 10 amino acid residues. Three samples isolated from the NK province had identical amino acid sequences. Nevertheless, three amino acids in major variable regions (155; T, 194; T and 236; D) and one amino acid in epitope D (155; T) differed from DuCV_CCS_KU002_22, which was previously classified as the same sub-genotype previously (Figure-4). Two isolates of group 2 had identical amino acid sequences, with nine amino acid residues on major variable regions (47H, 55N, 82R, 107T, 177I, 183I, 194G, 197H, and 236N) and each amino acid residue on A-D epitopes (55N, 82R, 107I, and 177I) differing from those of the NK isolates (Figure-4), of which several residues were identical to sub-genotypes Ic and Id (Table-3).

**Table-3:** Differences in amino acid residues among our isolates and reference sub-genotypes.

Isolate and reference subgenotypes	Amino acid position of cap protein																			

	47*		55*^†^		56*^†^	82^†^		104*^†^		106*^†^		107*^†^		109*^†^		131		154*^†^		155*^†^
DuCV_NK_KU003_22	N		S		Q	Q		T		S		K		F		N		S		T
DuCV_NK_KU005_22	•		•		•	•		•		•		•		•		•		•		•
DuCV_NK_KU001-22	•		•		•	•		•		•		•		•		•		•		•
DuCV_CCS_KU002_22	•		•		•	•		•		•		•		•		•		•		A
DuCV_SP_KU004_22	H		N		•	R		•		•		T		•		•		•		•
DuCV_RB_KU006_22	H		N		•	R		•		•		T		•		•		•		•
Sub-genotype Ia	•		S/N		•	•		S/T		N		•		F/Y		•		•		•
Sub-genotype Ib	•		•		•	•		•		•		•		•		•		l		•
Sub-genotype Ic	N/H		•		Q/T	R		•		•		T		F/N		N/S		•		•
Sub-genotype Id	N/H		•		•	Q/R		•		•		K/T		•		•		A		•

**Isolate and reference subgenotypes**	**Amino acid position of cap protein**																			

	160*^†^	177*^†^		182*			183*		194*		195*		197*		205*		236*		**246**	

DuCV_NK_KU003_22	I	V		F			V		T		T		Y		R		D		T	
DuCV_NK_KU005_22	•	•		•			•		•		•		•		•		•		•	
DuCV_NK_KU001-22	•	•		•			•		•		•		•		•		•		•	
DuCV_CCS_KU002_22	•	•		•			•		•		•		•		•		•		•	
DuCV_SP_KU004_22	•	I		•			I		G		•		H		•		N		•	
DuCV_RB_KU006_22	•	I		•			I		G		•		H		•		N		•	
Sub-genotype Ia	I/T	•		•			•		•		•		•		•		•		•	
Sub-genotype Ib	•	•		F/L			•		•		T/R		•		•		•		•	
Sub-genotype Ic	•	•		•			I		G		•		H		K		N		T/A	
Sub-genotype Id	•	•		•			•		G		T/S		•		•		N		•	

* represents amino acid residues in major variable regions. † represents residues in epitope regions. Black dots represent amino acids identical to those of DuCV_NK_KU003_22

## Discussion

Several members of the genus *Circovirus* mainly target immune organs [29]. Viral replication can induce lymphocytic destruction through apoptotic mechanisms, leading to immunosuppression in infected hosts [8, 9]. Yuan *et al*. [17] investigated coinfection by circovirus and other pathogens in various species. These include swine, canine, and wild carnivores, which can cause severe clinical diseases [29]. Therefore, the coinfected group exhibited significantly more severe clinical lesions and pathogenicity than the mono-infected group [3, 30, 31]. Concurrent infections and other factors associated with the severity of clinical outcomes and pathogenicity of circovirus infections. Like other circoviruses, DuCV is also an immunosuppressive virus that can make hosts prone to infection by various viruses and bacteria [32]. DuCV and Goose parvovirus coinfection synergistically cause poor growth performance, atrophy of immune organs, and increased severity of infections [14, 33].

Moreover, an experimental study on DuCV combined with APEC infection reported that these infections increased the degrees of pathogenicity in 1-day-old ducklings [3]. In this study, we first identified Thai DuCV isolates from meat and layer ducks. Moreover, our DuCV cases revealed the presence of co-infection with other duck diseases, including RA, *E. coli*, DVEV, fungal infection, and DTMUV. DVEV is a contagious viral disease in waterfowl due to an increased mortality rate of up to 100% and decreased egg production [34]. Extensive outbreaks of DTMUV have been reported in duck farms in Thailand. DTMUV causes a significant decrease in egg production and severe neurological signs, leading to high morbidity and mortality rates [35]. Therefore, DTMUV and DVEV are significant threats to the Thai duck industry. As reported in a recent study, DTMUV and DVEV were also found in ducks infected with DuCV; however, the synergistic pathogenesis of DuCV infection with these viruses remains unclear [4]. Furthermore, the host-pathogen interaction and cellular responses to these coinfections must be clarified. The obtained data will help us develop methods and vaccines to control DuCV infection.

In this study, ducks infected with DuCV had differing macroscopic and microscopic lesions due to the involvement of different duck pathogens. DuCV can spread through horizontal transmission through the fecal-oral route and vertical transmission, similar to other circoviruses. Because of our study, more than half of the Thai DuCVs were isolated from layer ducks. Traditional integrated rice-laying duck farming has poorer biosecurity than chicken farming. We hypothesize that the movement of younger laying ducks in several parts of rice cultivation areas may contribute to the spreading of DuCV in duck flocks in Thailand. However, epidemiological studies have yet to focus on DuCV circulating in Thailand; it urgently needs study for disease monitoring and control.

DuCV has been classified into three genotypes using phylogenetic analysis based on the complete genome and *cap* gene [7, 36]. Based on the whole genome and cap gene analyses of viruses isolated from layer and meat ducks in Thailand in 2022, this study revealed that genotype I was widespread among the duck farms, which experienced feather loss, lower-than-average weight, growth retardation, and poor body condition. The study also involved phylogenetic tree and genetic distance analyses. At the same time, several countries have reported that both genotypes I and II are circulating [25]. In Northern Vietnam, a country in Southeast Asia, genotypes I and II were found to be circulating in 2021, with 68.42% of the 38 farms being DuCV-positive farms [20].

Although positive samples from a variety of duck age ranges were reported, the age range of 5–7 weeks was significantly higher than that of the other age groups (<3 weeks and >77 weeks) [20]. Four of the six samples in this study, which ranged in age from 5 to 7 weeks, showed positive results. Two DuCV-positive samples, one from a meat duck and the other from a layer duck, were determined to be 3 and 28 weeks old, respectively. The Thai isolates provided nucleotide sequences of the complete genome spanning from 1995 to 1996 bp; however, previous research indicated variant lengths of 1987 to 1996 bp for genotypes I and II and 1755 bp for genotype III [7, 15, 20]. Different nucleotide sequences from our isolates were discovered in non-coding DNA, consistent with previous findings that no deletion or insertion mutation was discovered in the protein-coding regions of the genome [20]. Furthermore, all of our samples carried the conserved mononucleotide stem-loop motif (TATTATTAC), which is the source of known circovirus replication [15].

Phylogenetic patterns and genetic distances from the complete genome and *cap* gene revealed that Thai isolates were divided into two groups. Based on reference sub-genotypes that have been reported Ia-Id, first group was determined to be sub-genotype Ib with 98.22%–99.53% similarity of the complete genome and 97.36%–99.87% similarity of the *cap* gene, of which this sub-genotype was reported to be distributed in Vietnam and South Korea [20]. The second group comprised two viruses isolated from farms in two provinces with an ancestor node associated with the reference sub-genotype Id. However, the group’s similarity percentage to all reference sub-genotypes prevented them from being classified into sub-genotypic categories (Table-2). This sub-genotype may be a new sub-genotype of genotype I. The cap protein of Circovirus is the major antigenicity of the viral structural protein, which has been proposed in six major variable regions and five epitope regions [7, 20]. Amino acid alignment based on Cap protein among our isolates and reference sub-genotypes revealed 21 amino acid residues that differed among sub-genotypes. Thai isolates had 10 amino acid residues different within groups, of which 4 were located in major variable and epitope regions. An amino acid variant is essential for developing a specific serological diagnostic test kit or vaccine.

## Conclusion

The occurence of DuCV infection in ducks with a history of feather loss, underweight, growth retardation, and poor body condition was reported in this study. Six DuCVs had at least two sub-genotypes in genotype I. It is important to comprehend the genetic and amino acid differences in DuCV in these regions to perform molecular epidemiological investigations and develop preventative and control strategies. Nevertheless, this was the first study on DuCV conducted in Thailand, and the epidemiology of DuCV has not yet been investigated in duck-producing regions.

## Authors’ Contributions

SK and PL: Designed and conducted the study and wrote the manuscript. TS: Communicated with owners of duck farms, observed histopathological slides, and supervised the study. SP: PCR and RT-PCR methods. PS: Performed histopathological preparation. NS: Assisted in bacteriological study. RK: Sample collection. All authors have read, reviewed, and approved the final manuscript.
